# Role of PPARs and Retinoid X Receptors in the Regulation of Lung Maturation and Development

**DOI:** 10.1155/2007/91240

**Published:** 2007-06-27

**Authors:** Dawn M. Simon, Thomas J. Mariani

**Affiliations:** ^1^Division of Pulmonology, Allergy/Immunology, Cystic Fibrosis and Sleep, Department of Pediatrics, School of Medicine, Emory University, Atlanta, GA 30322, USA; ^2^Division of Pulmonary Medicine, Brigham and Women's Hospital, Harvard Medical School, Boston, MA 02115, USA

## Abstract

Understanding lung development has significant importance to public health because of the fact that interruptions in the normal developmental processes can have prominent effects on childhood and adult lung health. It is widely appreciated that the retinoic acid (RA) pathway plays an important role in lung development. Additionally, PPARs are believed to partner with receptors of this pathway and therefore could be considered extensions of retinoic acid function, including during lung development. This review will begin by introducing the relationship between the retinoic acid pathway and PPARs followed by an overview of lung development stages and regulation to conclude with details on PPARs and the retinoic acid pathway as they may relate to lung development.

## 1. THE RETINOIC ACID PATHWAY AND PPAR

The effects of retinoic acid are mediated by the retinoic acid receptors (RAR) and retinoid X receptors (or 9-cis retinoic acid receptor, RXR). RARs and RXRs each have 3 separate subtypes: *α*, *β*, and *γ*. RXR is specific for the 9-cis retinoic acid (9CRA) stereoisomer, while RAR binds both 9CRA and all-trans retinoic acid (ATRA). RARs form heterodimers with the three RXR subtypes and RXR's form heterodimers with members of the nuclear receptor family, including PPAR*γ*. RXRs can also form homodimers, which among other effects, can activate PPAR target genes [1]. While as a group, the three PPAR isoforms (*α*, *β*/*δ*, and *γ*) function to regulate cellular lipid utilization and homeostasis, each isoform has discrete yet overlapping functions and ligand specificities. Upon activation by an appropriate ligand, PPARs form an obligate heterodimer with RXR to recruit nuclear receptor coactivators. Because they function as heterodimers with the RXR, PPARs could be considered an extension or modulator of the retinoic acid signaling pathway. The canonical pathway is that these ligand-activated PPAR-RXR heterodimers bind to peroxisome proliferator response elements (PPREs), and activate gene transcription, although PPARs can also serve as active transcriptional repressors [2]. Furthermore, nongenomic functions of PPARs upon gene regulation (e.g., regulatory effects independent of PPRE binding) have been reported [3–5]. For instance, PPARs are capable of trans-repression of other transcription factors, through direct interaction or through interaction with other coactivator/corepressors. Schupp and colleagues recently demonstrated that a RAR*α* antagonist can directly affect PPAR*γ* activity and therefore be considered both a PPAR*γ* agonist and RAR*α* antagonist [6]. Additionally, Szatmari and colleagues found that PPAR*γ* regulates CD1d, a molecule involved in dendritic cell antigen presentation, by inducing retinoic acid synthesis through RAR*α* [7]. These observations highlight the complex interplay between nuclear receptors. Given the numerous pathways through which PPARs could regulate gene expression either directly or indirectly, it is easy to envision that they may play a role in the complex regulatory mechanisms of lung development.

## 2. THE REGULATION OF MAMMALIAN LUNG DEVELOPMENT

Mammalian lung development follows a highly regulated, morphogenetic program beginning near mid-gestation and continuing through postnatal life [8, 9]. The mammalian lung initiates as an out-pouching of the ventral foregut endoderm. Initially, during the “embryonic” stage of organ development, which occurs during 5th and 6th week of gestation in the human or embryonic days 9.5 (E9.5) and E10.5 in the mouse, the lung arises as a ventral diverticulum of the foregut endoderm, separating from the esophagus and elongating caudally. This bud branches to give rise to the main bronchi of the left and right lung. Significant recent advances have been made in the understanding of the genetic and molecular mechanisms governing many of the early processes of lung development [10, 11]. Lung bud initiation and outgrowth is controlled by both the Gli/Shh pathway [12–14] and FGF receptor signaling [15].

Beginning in the pseudoglandular stage (which occurs between 6 and 16 weeks of gestation in humans or E10.5–16.5 in mice) and continuing through the canalicular stage (which occurs between 16 and 26 weeks of gestation in humans or E16.5–17.5 in mice), this lung bud subsequently undergoes repeated rounds of dichotomous branching to produce the tree-like structure of the mature conducting airway. Numerous molecules are currently appreciated as playing a role in the branching process. Many, though certainly not all, of these molecules belong to the BMP and FGF signaling pathways [16–21]. BMP-4 and FGF-10 are believed to form signaling centers that specify branch initiation sites and outgrowth [22]. Locations of branching specificity are limited, in part, by molecules such as Sprouty and Noggin, which antagonize FGF and BMP signaling [23, 24]. Many other factors such as EGF, Shh, and Wnt also play a role in the regulation of branching morphogenesis. The involvement of these particular pathways also highlights the role of epithelial-mesenchymal interactions in lung development. It is well accepted that epithelial-mesenchymal interactions are essential for normal lung development, primarily during embryonic growth and differentiation [25, 26]. The specific role of epithelial-mesenchymal interactions in later stages of lung development, including postnatal lung maturation, is unclear. In addition to the continuation of branching morphogenesis, the canalicular stage is marked proximo-distal cell type specification and vascularization.

From 26 to 36 weeks of gestation (E17.5 through postnatal day 4 in mice), the “saccular” stage completes formation of the conducting airway tree and differentiation of distal epithelial cells. During this stage, the distal architecture of the lung dramatically changes due to further differentiation and flattening of distal airway epithelia. This process is coordinated by factors such as GATA-6, Nkx2.1, HNF3*β*, C/EBP*α*, glucococorticoid hormones, and FGFs [27]. At or near the end of the saccular stage, the lung becomes prepared for a transition to air breathing with the production of pulmonary surfactant. Recent studies support a role for the forkhead box transcription factor, Foxa2 as a master regulator of surfactant production [28], in coordination with the transcription factors, Ttf1 and C/EBP*α* [29]. The calcineurin/NFAT signaling pathway also appears to play a role in this process [30].

Finally, the gas exchange portions of the lung are formed during the alveolar stage of development. This occurs beginning in week 36 of human gestation and continues through early childhood. In mice, this stage occurs entirely during the postnatal period, beginning in the first week of life and continuing through the first month. Maturation of gas-exchange capacity involves airway wall secondary crest septation and elongation, a process referred to as alveogenesis. Elongation of secondary septae results in partitioning of saccules into alveolar ducts and alveoli with an increase in gas-exchange surface area. Lung maturation and alveogenesis continues after birth in both rodents and humans. Although the number of airway generations and branching pattern of the lung is established at birth, the morphology of the lung parenchyma is quite different between the newborn and the adult [31]. Alveoli continue to form for at least 2 years after birth in humans. A detailed understanding of the regulatory processes controlling alveogenesis is lacking. Retinoic acid (discussed further below), PDGF, and FGF signaling all contribute to the regulation of secondary crest elongation. PDGF-A is essential in alveolar formation as defined by failed alveogenesis in its deficiency state secondary to a lack of development of alveolar myofibroblasts [32]. FGF signaling is also critical to alveogenesis, again, as defined by combined deficiency in FGFR3 and FGFR4 [33]. Interestingly, the ligand(s) mediating this effect is unknown. These data can be integrated into a model predicting morphogenic gradients of RA and FGF signaling secondary crest elongation [34]. In recent years, the importance of coordinated development of the vasculature during alveolarization has gained appreciation. It is clear that the appropriate balance of VEGF activity, which is an important pathway for vascular development and maintenance, plays a critical role in alveogenesis [35–38]. VEGF also appears to play a critical role in promoting surfactant expression [39].

Boyden and Tompsett have described a mechanism for airspace formation distinct from the process of saccule subdivision by secondary septal elongation; the transformation of terminal or respiratory bronchioles into alveolar ducts [31, 40]. Massaro et al. corroborated this concept, finding that airspaces can develop through the nutritionally-dependent elongation of the conducting airway and de novo formation of alveoli (termed “retrograde alveolarization of bronchioles”) [41]. Since the time of these seminal observations, only a few studies have clarified the regulation of alveolar duct formation and its contribution to airspace structure. Intact collagen and/or elastin fibers appear necessary for the development of alveolar ducts, as treatment of neonatal rats with the BAPN, an inhibitor of the collagen and elastin cross-linking enzyme lysyl oxidase results in increased volume density of alveolar ducts [42]. Indomethacin treatment of neonatal rats also results in increased alveolar duct formation, implicating endogenous prostaglandin levels as a regulatory component in this process [43].

## 3. RETINOIC ACID SIGNALING IS ESSENTIAL AT MANY POINTS IN LUNG DEVELOPMENT

**Figure 1:**
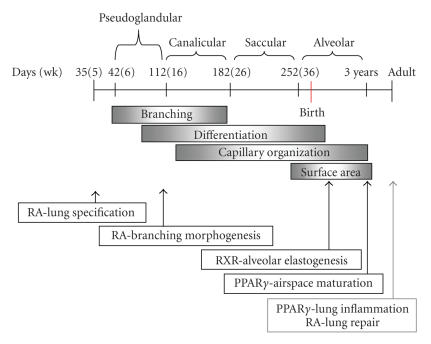
*Retinoic acid and PPARγ signaling are essential at many points during lung development.* Lung development occurs in multiple stages (top), each involving critical processes (middle) and multiple regulatory factors. This schematic highlights the timeline for human lung development, though murine lung development occurs in similar stages. It is widely appreciated that retinoic acid signaling has effects on all stages of lung development (bottom). Recently, PPAR*γ* has also been found to be a critical modulator of postnatal lung development. (Adapted from Mariani, T.J. Developmental genetics of the pulmonary system. In: Moody, S.A., Editor, Principles of developmental genetics. Burlington, VT: Academic Press, 2007:932-945. With the permission of Elsevier Inc.)

The retinoic acid pathway can have effects on all stages of lung development (see Figure 1). The RARs and RXRs have distinct expression patterns, notably during mouse embryonic development [44–47]. Specifically, RXRs have been shown to be expressed in the human lung during critical periods in development from 13 weeks gestation until term, then their expression becomes markedly reduced in the adult [44]. Interestingly, retinoic acid signaling is downregulated during lung epithelial tubule branching and differentiation, which ultimately allows formation of mature type I and II cells [46, 48].

To understand their functional role, gene-targeted mice have been generated for all 3 RARs and RXRs [49–51]. RAR single mutants are viable though they display a range of vitamin A deficiency syndromes, which increase when double null mutants are generated [49, 50]. RXR*α* loss results in fetal lethality at around E14.5 [52, 53]. Similar to PPAR*γ* null mutants, these mice display severe myocardial hypoplasia. Because of the in utero lethality, mice with alleles for conditional gene targeting have been generated [51]. Based on these studies, RXR*α* has been found to be a crucial mediator of metabolism and skin development [54–57]. RXR*β* mutant fetuses also have high mortality (50%) with infertility in viable male pups [58]. RXR*γ* mutant mice survive and are fertile though they have abnormal metabolism secondary to alterations in pituitary-thyroid axis [59, 60]. The development of these genetically altered mice has provided insight into the functional role of the RA signaling pathway as it relates to lung development. Targeted deletion of RAR*β* alters the regulation of lung septation [61]. RAR*γ* deletion also results in reduced elastic tissue and alveolar number with increase in mean chord length [62]. The authors found similar results with RXR*α* deletion. Desai and colleagues demonstrated that balanced activation of RAR*α* and *β* is critical for normal lung bud initiation and endodermal differentiation [63]. Mollard and colleagues determined that RA signaling through RAR*β* during the pseudoglandular stage promotes the formation of conducting airways [64]. Because single RAR mutants have few to no lung abnormalities [61, 64–68], double mutants have been developed because of the apparent redundancy in these receptors. For example, RAR*α*/RXR*α* and RAR*α*/*β* double mutants develop lung hypoplasia or agenesis [69–71]. Additionally, retinoids are capable of promoting the formation of alveoli in neonatal rats and in adult rats with elastase-induced emphysema [72, 73].

## 4. EPITHELIAL CELL PPAR*γ* EXPRESSION CONTRIBUTES TO THE REGULATION OF LUNG MATURATION

Most of the literature regarding the role of PPARs in the lung has focused on understanding PPAR*γ*. While PPAR*α* shares the common characteristic of having potent anti-inflammatory properties with PPAR*γ*, it has not been shown to have a role in regulating lung development. Similarly, there has been no description for a role of PPAR*β*/*δ* in modulating lung development though Matsuura and colleagues demonstrated upregulation of PPAR*β*/*δ* expression in induced human tracheobronchial epithelial (HBE) cells which suggests that PPAR*β*/*δ* may have a role in the squamous differentiation process of airway cells [74]. PPAR*γ* is expressed as at least 2 different isoforms, *γ*1 and *γ*2. These isoforms differ only by the addition of 30 amino acids at the amino terminus of *γ*2, and appear to be functionally equivalent. While PPAR*γ*2 is expressed primarily in adipose tissue, PPAR*γ*1 is expressed in a broad range of tissues including the lung, heart, skeletal muscle, large and small intestine, kidney, pancreas, spleen, and breast [5, 75]. Within the lung, PPAR*γ* expression has been reported in the airway epithelium [76, 77], bronchial smooth muscle [76, 78], endothelial cells [79], macrophages [80], eosinophils [81], and dendritic cells [82]. There is little data describing the expression of PPAR*γ* in the developing lung. Barlier-Mur and colleagues found that PPAR*γ*1 mRNA was detectable at 18 days gestation in fetal rat lungs, as well as the C/EBPs [83]. The expression of these factors increased during development, peaking just prior to delivery. While they and others [84] have reported PPAR*γ* expression in type II alveolar cells, they did not see that this expression pattern was developmentally regulated, although it could be induced by exposure of cultured type II alveolar cells to dexamethasone, retinoic acid, EGF, and KGF. Interestingly, PPAR*γ* protein concentrations were only induced by KGF, and not with EGF or dexamethasone. We observed a spatial and temporally restricted pattern of PPAR*γ* expression, including prominent immunolocalization within the conducting airway epithelium of normal mouse lungs [85]. This pattern of staining was first detectable at birth and increased in intensity over the first few weeks of life in mice.

PPAR*γ* can play a prominent role in regulating cellular differentiation. PPAR*γ* is sufficient and necessary to promote the formation of adipocytes and the development of adipose tissue in vivo [75, 86]. This appears to be due, at least in part, to the ability of PPAR*γ* to regulate numerous genes involved in lipid metabolism. Complete germ-line PPAR*γ* deficiency in mice results in embryonic death at mid gestation, prior to lung development due to failed placental cytotrophoblast differentiation, which is necessary for placental vascularization [87]. Recently, Duan and colleagues generated a mouse model of complete PPAR*γ* deficiency that spared the trophoblast, allowing delivery of viable pups that they used to study the role of PPAR*γ* in the metabolic syndrome [88]. Unfortunately, there was no description of effect on lung development. A role for PPAR*γ* in promoting cellular differentiation is also suggested by its antitumor effects in vivo and in vitro, which include suppressing cellular proliferation, promoting cell death, and inducing differentiation of malignant tumors cells from various organs including the lung [89], breast [90], colon [91], and adipose tissue [92]. In isolated lung epithelial cells, PPAR*γ* can promote the expression of markers for terminal differentiation including the expression of surfactant associated protein genes [93–95].

In addition to its roles in cellular differentiation and organ/tissue development, PPAR*γ* is widely appreciated as a regulator of tissue inflammation, which will be discussed in other sections of this review. In brief, PPAR*γ* activation can modulate various immune cell functions. For example, PPAR*γ* regulates monocyte/macrophage differentiation and promotes cellular activation as measured by increased production of metalloproteinases and reactive oxygen species [96]. Dendritic cells express PPAR*γ*, which upon activation can influence cell maturation and antigenic peptide presentation to T cells [82, 97]. PPAR*γ* is expressed at low levels in resting T cells, but is increased following T cell activation where PPAR*γ* can then inhibit T cell IL-2 and IFN*γ* production [98]. Additionally, PPAR*γ* activation has an antiproliferative and cytotoxic effect on normal and malignant B cells [99]. While PPAR*γ* expression has been reported in these various cell types, the target cells and mechanisms for the protective, anti-inflammatory activities of PPAR*γ* ligands within the lung are unclear. Some of these inflammation-related functions of PPAR*γ* appear to mediate, at least in part, the regulation of resident cell functions. PPAR*γ* has been shown to be expressed in cultured human airway smooth muscle cells and its activation inhibits cell growth while inducing apoptosis and inhibits release of GM-CSF and G-CSF to a greater extent than dexamethasone, a medication frequently used in asthma [78]. Further, in cultured human airway epithelial cells, PPAR*γ* activation can inhibit expression of proinflammatory mediators such as TNF-*α*, IL-8, iNOS, and MCP-1 [5, 77, 81].

Our laboratory sought to understand the physiological role of epithelial cell PPAR*γ* and its potential contribution to lung development and homeostasis, considering the fact that PPAR*γ* is capable of having a significant and complex influence upon cellular differentiation, organ development, and the control of tissue homeostasis. We hypothesized that epithelial cell PPAR*γ* might be necessary for the establishment and maintenance of normal lung structure through regulation of epithelial cell differentiation and/or control of lung inflammation.

**Figure 2:**
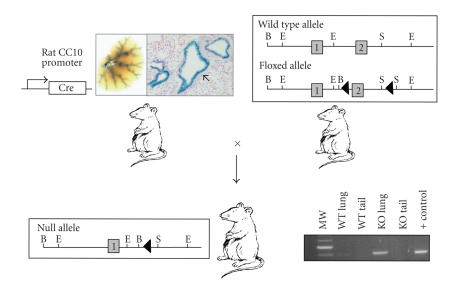
*The generation of conditionally targeted epithelial cell PPARγ deficient mice [85].* We developed a line of mice capable of targeting the airway epithelium by expressing Cre recombinase under the direction of the rat CC10 promoter (top, left). These mice, termed CCtCre, were crossed with the ROSA26 Cre reporter mouse to test the efficiency for recombining loxP sites in vivo which demonstrated *β*-galactosidase staining limited to the conducting airway epithelium (arrow within inset). We crossed the CCtCre mice with mice homozygous for a PPAR*γ* allele with a pair of loxP sites flanking exon 2 of the gene (top, right) [100], creating mice with PPAR*γ* deficiency limited to the conducting airway epithelium (bottom, left). The conditional targeted genotype was confirmed by identification of gene rearrangement specifically in the lung alone (bottom, right).

Using a conditional targeting strategy, we deleted the PPAR*γ* gene specifically within conducting airway epithelial cells [85]. We started by generating a new line of Cre Recombinase-expressing targeting mice, termed CCtCre, where the rat CC10 promoter was used to drive Cre expression specifically within the lung conducting airway epithelium. Functional targeting specificity in these CCtCre mice was confirmed by crossing them to the ROSA26 reporter line. Crossing the CCtCre mice with mice engineered to have loxP sites (targets of Cre-mediated recombination) flanking exon 2 of the PPAR*γ* gene led to targeted deletion within the airway epithelium (see Figure 2).

Lungs from PPAR*γ* conditionally targeted, airway epithelial cell PPAR*γ* deficient mice revealed structural and functional abnormalities at maturity, but not prior to maturity, including enlarged airspaces consistent with a deficiency in postnatal lung maturation (see Figure 1). Abnormal airspace structure persists throughout adulthood, but is not progressive and occurs in the absence of inflammation. While control animals show a reduction in mean airspace size between 2 and 8 weeks of age, conditionally targeted, airway epithelial cell PPAR*γ* deficient animals do not. These data suggest that the phenotype results from an insufficiency in postnatal lung maturation. This does not appear to be the result of a defect in alveogenesis, as numerous normal-sized alveoli exist in conditionally targeted lungs. However, an abnormal distribution of airspaces, with increased numbers of alveolar ducts is observed (unpublished observations).

No qualitative or quantitative changes in the major classes of airway and airspace epithelial cells are evident, but some characteristics of airway epithelial cell differentiation appear affected. We found, through genome wide expression analysis of targeted airway epithelial cells, changes consistent with alterations in PPAR*γ* function (Lip1, Abca1, and Apoe) and cellular differentiation (Moesin, Ctsb, Klf13). We believe that altered epithelial-mesenchymal interactions, secondary to epithelial PPAR*γ* deficiency, lead to changes in extracellular matrix gene expression and abnormal lung structure at maturity. Efforts to further define the mechanism(s) mediating this abnormality and to test the role of this transcription factor in regulating airway inflammation are the focus of current investigation.

In summary, it is well appreciated that the retinoic acid signaling pathway contributes to the regulation of lung development at many different stages, including during terminal maturation giving rise to the functional gas exchange units of the lung, the alveoli. Although retinoic acid activity during alveogenesis appears to be linked to elastin fiber formation, the cellular and molecular mechanisms for these effects are not well defined. It has recently become apparent that PPAR*γ* has a role in contributing to these regulatory processes. Again, the mechanisms at work are yet to be defined. Potentially, they involve the regulation of epithelial cell differentiation, and may act in part through interaction with the RARs and RXRs. Tremendous current activities in the field of PPAR biology should rapidly lead to a better understanding of the role of these transcription factors in promoting lung maturation and their potential contribution to human lung disease.
